# Influence of Body Position on Cortical Pain-Related Somatosensory Processing: An ERP Study

**DOI:** 10.1371/journal.pone.0024932

**Published:** 2011-09-15

**Authors:** Chiara Spironelli, Alessandro Angrilli

**Affiliations:** 1 Department of General Psychology, University of Padova, Padova, Italy; 2 CNR Institute of Neuroscience, Padova, Italy; Royal Holloway, University of London, United Kingdom

## Abstract

**Background:**

Despite the consistent information available on the physiological changes induced by head down bed rest, a condition which simulates space microgravity, our knowledge on the possible perceptual-cortical alterations is still poor. The present study investigated the effects of 2-h head-down bed rest on subjective and cortical responses elicited by electrical, pain-related somatosensory stimulation.

**Methodology/Principal Findings:**

Twenty male subjects were randomly assigned to two groups, head-down bed rest (BR) or sitting control condition. Starting from individual electrical thresholds, Somatosensory Evoked Potentials were elicited by electrical stimuli administered randomly to the left wrist and divided into four conditions: control painless condition, electrical pain threshold, 30% above pain threshold, 30% below pain threshold. Subjective pain ratings collected during the EEG session showed significantly reduced pain perception in BR compared to Control group. Statistical analysis on four electrode clusters and sLORETA source analysis revealed, in sitting controls, a P1 component (40–50 ms) in the right somatosensory cortex, whereas it was bilateral and differently located in BR group. Controls' N1 (80–90 ms) had widespread right hemisphere activation, involving also anterior cingulate, whereas BR group showed primary somatosensory cortex activation. The P2 (190–220 ms) was larger in left-central locations of Controls compared with BR group.

**Conclusions/Significance:**

Head-down bed rest was associated to an overall decrease of pain sensitivity and an altered pain network also outside the primary somatosensory cortex. Results have implications not only for astronauts' health and spaceflight risks, but also for the clinical aspects of pain detection in bedridden patients at risk of fatal undetected complications.

## Introduction

Pain is a complex subjective experience arising from the integration of several multi-dimensional aspects, ranging from sensory to cognitive to affective-motivational domains, all of which contribute to mark the pain experience. In electrophysiological studies, electrical surface stimulation elicits early somatosensory evoked potentials with peak latencies of less than 80 ms, shown to reflect the earliest brain responses to incoming somatosensory information [1]). The subjective distinction from somatosensory changes and painful sensation is possible only considering late cortical potentials with latencies ranging from 80–100 to 500 ms [1], [2]. These late components are modulated by secondary cortical mechanisms which account for all phases of painful/painless stimulus processing, from the detection of its basic characteristics in somatosensory cortices (SI-SII), such as stimulus recognition, intensity estimation, etc., to top-down cognitive aspects of pain detection, such as memory, vigilance, attention and distraction [1], [3]. The delayed cortical distinction between noxious/painful and innocuous/painless stimuli is the direct consequence of the physiological activation of different peripheral fibers, since large-diameter, fast-conducing (30 to 60 m/s in man) myelinated Aβ axons mediate non-nociceptive input, whereas thin myelinated Aδ (4 to 30 m/s) and unmyelinated C fibers (0.4 to 1.8 m/s) convey noxious stimuli [e.g.], [ 3,4].

Functional imaging studies were able to localize the complex neural network underlying the two main dimensions of pain, i.e., the sensory discriminative and the affective-motivational components [5]. Such a network includes the somatosensory (SI-SII) and insular cortices, contralateral to stimulation site, the anterior cingulate, the amygdala and several subcortical nuclei [6]–[10]. Several conditions, physiological and psychological variables are able to influence pain perception (e.g. attention, emotion, etc. [11]–[13].

A novel condition which may influence pain processing is simulated microgravity: it consists of Head-Down Bed Rest (HDBR) position, in which gravity force is orthogonal to the cephalic-caudal axis, and which represents both physiologically and perceptually the ground position best resembling weightless space condition [14]. Past studies demonstrated that both during spaceflight and its analogue, prolonged HDBR, almost all physiological processes are significantly altered, leading to, e.g. cardiovascular deconditioning, bone and muscle mass loss, fluid shifts towards upper body, hormonal changes, enhanced calcium excretion, etc. [15]–[18]. Concerning cortical activity, there is an ongoing debate as to whether HDBR position induces a general alteration of cognitive functioning or rather a selective inhibition of a few specific and complex perceptual/cognitive abilities. Indeed, past studies showed that HDBR elicited an increase of low-frequency EEG rhythms, i.e. the delta and theta bands [19], [20], indexes of cortical inhibition which suggest a possible cognitive impairment induced by microgravity. In addition, Schneider and colleagues [21] found high beta EEG band (18–35 Hz) with unaffected amplitude in the normal gravity condition, followed by a significant inhibition of beta rhythm in the weightlessness condition, a result which has been interpreted to be mainly related to emotional activation/anxiety. A review of the available records in the space (typically carried out with very few participants and therefore characterized by a very low statistical power) revealed an impairment mainly in complex cognitive tasks, e.g., visual-motor tracking and dual-task performance [22]. Other studies found no clear effects of microgravity on sleep or vigilance. However, in ground studies characterized by better statistical power, HDBR induced mild deterioration of high attention functioning in the morning [23] and, during parabolic flight, increased cortical beta EEG power was found, but this had no effect on participants' performance in a motor tracking task [24]). In any case, while the influence of weightless on visual perception and motor coordination have been relatively more investigated in past studies, data on the effects of microgravity on somatosensory functions and pain, to our knowledge, are lacking. In line with the main classification of physiological changes induced by weightlessness, which divides events in short-term (seconds), middle-term (several minutes to days) and long-term (weeks to months) [25], we adopted a middle-term duration of 90 min simulated microgravity.

Starting from past observations on HDBR [19], [20], the present study hypothesized that simulated microgravity significantly dampens pain perception and reduces cortical processing of painful electrical stimuli.

## Materials and Methods

### Participants

Twenty post-graduated healthy men were recruited from the “*Centro Interdipartimentale di Studi e Attività Spaziali – CISAS*” (i.e., Center of Studies and Activities for Space) of Padova. All participants were male, post-doc researchers, a sample matching astronauts' socio-educational characteristics. Subjects were free of cardiovascular or other medical pathologies, and denied drug therapy at the time of the experiment. Every participant was requested to avoid smoking and coffee drinking before the experimental session, which started at 9 o'clock in the morning. Subjects were randomly assigned to the experimental condition (i.e., the Head-Down Bed Rest position, BR) or the control group (i.e., the sitting position). Groups were comparable for age (BR mean: 29.5±5.46 years; Control mean: 30.0±6.0 years; *t*(1,18) = 0.85, *NS*) and educational level (all participants had the Ph.D. degree). Subjects were on average 95% right-handed, according to the Edinburgh Handedness Inventory [26], and they were paid 54 € for the complete experimental session.

All participants gave their written informed consent to the study, that conformed the standard set by the Declaration of Helsinki. Experimental procedures were approved by the Ethics Committee of the Faculty of Psychology, University of Padova (Italy).

### Stimuli, task and procedure

**Figure 1:**
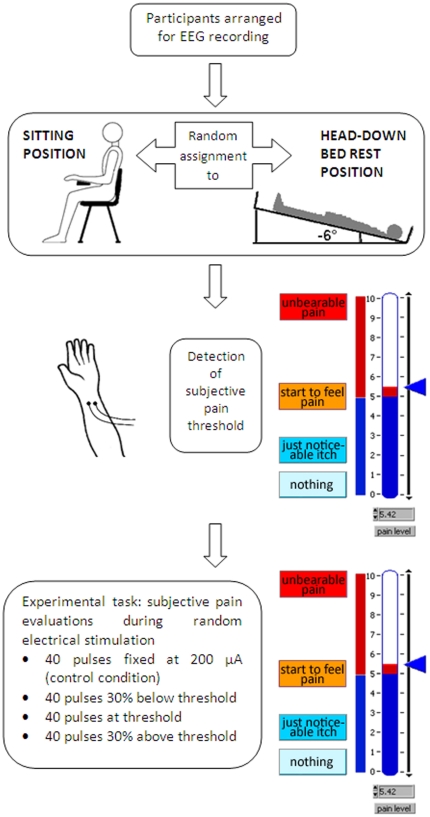
Schematic diagram of experimental design and procedure. After EEG preparation (top row), participants were randomly assigned to sitting or bed rest position (control and experimental group, respectively; second row). Two surface gold electrodes were applied on the internal left wrist and participants' electric pain threshold was computed by means of a 10-point visuo-analogue scale representing different levels of pain intensity (third row). Participants started the experimental task which consisted in the EEG recording and subjective pain evaluations during pseudo-random administration of four different levels of electrical intensities (bottom row).

Participants were familiarized with the equipment and procedure used. They were arranged for electrophysiological recording and randomly assigned to the BR or control sitting position (Fig. 1). Subjects in bed rest condition laid with head down on a mattress inclined -6 degrees, a standard condition of simulated microgravity named Head Down Bed Rest (HDBR): this position has been evaluated by astronauts as the best which resembles perceptually the weightless condition in space. This condition has proven to induce physiological changes very similar to those measured in space [14]. A PC laptop was stably placed 40 cm above body to allow bed rest participant to perform pain evaluations. The control group was sitting during the whole experiment on a chair in front of the same PC laptop used for BR. After 90 minutes of bed rest/sitting condition, in which experimental requests were presented and qualitative interviews for state/trait anxiety assessment (STAI-Y1 and STAI-Y2, respectively; Italian version by [27]) were administered, the experimental session started.

For electric stimuli, two surface gold electrodes were applied on the internal left wrist, near the hand joint (Fig. 1): in particular, we paid attention to ensure that electrodes were applied orthogonally to the finger superficial flexor muscle, and not on median and ulnar nerves or finger flexor tendons, by ascertaining that no involuntary finger movements were elicited by electrical pulses. The electric pain threshold was computed for every participant by means of a LabVIEW (National Instruments, Texas) *ad hoc* program which administered electrical stimuli with increasing current intensity. The participants' pain threshold was tracked with a method derived from the adaptive procedure, the simple up-down staircase [28], [29] which tracks the 50% of the psychometric function. Electrical stimuli were delivered by a battery powered constant current stimulator controlled by PC through the parallel port. The first electrical pulse was very weak (39 microAmperes, µA) and typically was not detected by the participant, but stimulus intensity was progressively increased using an ascending method of limits with current increments ranging randomly between 39 and 234 µA until the subjective threshold pain was reached. Every electrical pulse lasted 10 milliseconds. Subjects had to evaluate every electric pulse using a 10-point visuo-analogue scale representing different levels of pain intensities (Fig. 1). The pain threshold procedure stopped when the average pain perceived in five consecutive electric pulses surpassed the pain threshold corresponding to the critical level of 5 (labelled “I start to feel pain”; see Fig.1). The interval between the end of an evaluation and the beginning of the next one varied randomly between 3 and 4 seconds. In this experimental setting, the Inter-Stimulus Interval (ISI) corresponds to the Inter-Trial Interval (ITI). After the pain threshold procedure finished, the program computed an on-line regression coefficient of the current/subjective evaluation sequence for precisely determining pain threshold. The regression line allowed to compute the interpolated exact current intensity in µA, corresponding to the subjective pain threshold rating 5.

Soon after pain threshold assessment, participants started the experimental task consisting in EEG and subjective pain evaluation recording during a pseudo-random electrical stimulation administration (similarly to pain threshold evaluation, each electrical pulse lasted 10 ms). Four different levels of electrical intensities were administered: since past evidence [e.g. 30] revealed lower arousal levels in supine compared with stranding position, we introduced a control, painless and often undetected condition of 200 µA, fixed for all participants to ensure that both groups had similar levels of cortical activation in a virtual “baseline condition”; in addition, three electrical stimuli levels were computed starting from subjects' individual pain thresholds. Therefore, the program generated, pseudo-randomly interspersed, (1) forty Under Threshold electrical pulses, corresponding to 30% reduced electrical current level (i.e. 30% below subject's pain threshold), (2) forty Threshold pulses, corresponding to the electrical pain threshold, and (3) forty Over Threshold electrical pulses, corresponding to 30% incremented pain level (i.e. 30% above participants' pain threshold). Thus, two intensities were below (Control and Under Threshold) and two in the range of individual pain thresholds (Threshold and Over Threshold), in agreement with Bromm's recommendations [2]. Participants received a total of 160 electric stimuli (40 for each condition) distributed, across conditions, in a pseudo-random way, since we forced the program to present the same condition no more than 2 consecutive times, with a maximum of 5 repetitions of identical stimuli for each condition. This constraint, together with an ISI/ITI randomly varied between 3 and 4 seconds, allowed us to limit possible repetition suppression effects due to habituation [e.g. 31], [32].

At the end of pain evaluation, a qualitative interview for state anxiety assessment (STAI-Y1) was administered.

### Data recording and analysis

EEG cortical activity was recorded by 38 tin electrodes, 31 placed on an elastic cap (Electrocap) according to the International 10–20 system [33]; the other 7 electrodes were applied below each eye (Io1, Io2), on the two external canthi (F9, F10), nasion (Nz) and mastoids (M1, M2). All cortical sites were on-line referred to the left mastoid (M1). Data were stored using the acquire software NeuroScan 4.1 version. Amplitude resolution was 0.1 µV; bandwidth ranged from DC to 100 Hz (6 dB/octave). Sampling rate was set at 500 Hz and impedance was kept below 5 KΩ.

**Figure 2:**
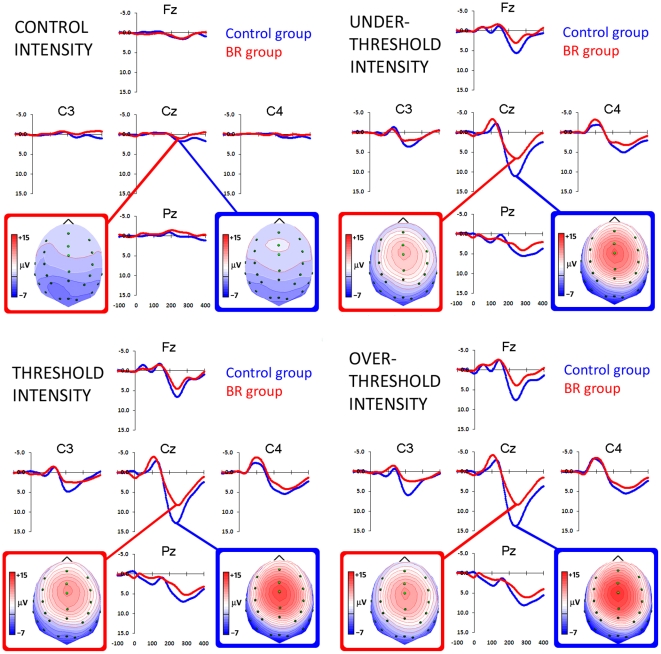
Group-level grand-average waveforms of selected electrodes (in correspondence of the somatosensory cortex) showing the time-course of somatosensory processing (top row) during Control and Under Threshold conditions (left and right panel, respectively), and pain processing (bottom row) during Threshold and Over Threshold conditions (left and right panel, respectively) in sitting controls (blue line) and Bed Rest participants (red line). Negativity is displayed upward. Spline interpolated maps of potentials representing scalp top views of P2 component (190–220 ms) in the four different conditions are depicted in blue and red boxes for control and Bed Rest groups, respectively.

EEG was continuously recorded in DC mode and stored for following analysis. Data were off-line re-referenced to the average reference, and a 40 Hz low-pass filter (no phase shift) was applied. After filtering, electrophysiological data were epoched into 1.2-s intervals, divided into 200 ms before and 1 s after stimulus onset. A 100-ms baseline preceding every electric pulse was subtracted from the whole trial epoch. Single trials were corrected for eye movement artifacts, i.e., vertical and horizontal movements, and blinking. BESA software (Brain Electrical Source Analysis, 5.1 version) was used to compute ocular correction coefficients, according to Berg and Scherg [34], [35]. Each trial was then visually inspected for any residual artifacts: overall, 27.3% of trails were rejected (Sitting Control group: 27.2%, 26.5%, 25.5% and 31.5%; BR group: 26.5%, 30.5%, 31.5% and 32.75%, for Control, Under Threshold, Threshold and Over Threshold intensities, respectively; the between-groups t tests were not significant). In line with ERP guidelines [36], we preferred to analyse time intervals rather than peak amplitude to avoid noise and arbitrary choices. In order to contrast different components of pain-related somatosensory processing, on the basis of visual inspection of grand average waveforms (Fig. 2) three functional time intervals known to correspond to specific phases of somatosensory and pain detection were chosen for data analysis, i.e., the two early components peaking around P1 (40–50 ms) and N1 (80–90 ms), and the late P2 component (190–220 ms). In agreement with other studies which investigated ERPs in pain evaluation [e.g. 1]–[3], [37], we referred to P1 and N1 components as the early indices of perceptual operations that are closely related to exogenous (i.e., stimulus-evoked) factors and that reflect the first brain responses to incoming somatosensory information and the automatic detection in the primary somatosensory cortex (S1) of the stimulated afference [3]. Conversely, we considered the P2 wave as an index that is related to pain-related endogenous factors and which reflects different late cognitive processing, such as pain localization or estimation [38], [39].

STAI-Y1 and STAI-Y2 anxiety scores were compared between groups by means of two-tailed *t* test as no differences on anxiety levels were expected (mean plus Standard Deviations [± SD] were reported). In addition, STAI-Y1 scores obtained before and after the whole experimental procedure were analyzed by means of repeated-measures analysis of variance (ANOVA), by comparing the between-groups factor Group (two levels: Sitting control vs. Bed Rest) and the within–subjects factor Time (Before vs. After experimental task). Subjective pain evaluations were compared between groups by means of one-tailed *t* test for the two pain intensity levels (i.e., Threshold and Over Threshold). The *t* test was computed one-tailed as worse, i.e., increased pain threshold was expected for BR group, and mean plus Standard Deviations [± SD] were reported.

Electrophysiological components of pain-related somatosensory processing were evaluated by means of analysis of variance (ANOVA) comparing, for each time interval, the average amplitude measured in four groups of electrodes, corresponding to regions of interest. On the basis of bioimaging studies of cerebral regions activated by painful stimulation and after visual inspection of grand-average waveforms (Fig. 2), four clusters (comprising the average activity of four electrodes) were selected: Lateral Left (LL: FT7, T7, TP7, P7), Medial Left (ML: FC3, C3, CP3, P3), Medial Right (MR: FC4, C4, CP4,P4), Lateral Right (LR: FT8, T8, TP8, P8). The between-subjects factor Group (two levels: Sitting control vs. Bed Rest) and two within–subjects factors entered ANOVA: Intensity (four levels: Control vs. Under Threshold vs. Threshold vs. Over Threshold) and Laterality (four levels: Lateral Left vs. Medial Left vs. Medial Right vs. Lateral Right regions). The Huynh–Feldt (HF) correction was applied where sphericity assumptions were violated [40]; in these cases, the uncorrected degrees of freedom, epsilon values and the adjusted *p* values have been reported. Post-hoc comparisons were computed using the Newman-Keuls test and statistical significance was expressed at the *p*< 0.05 level.

In order to identify brain regions activated during the time intervals of interest (corresponding to P1, N1 and P2 components), source localization was computed by means of standardized Low-Resolution Brain Electromagnetic Tomography (sLORETA [41]). Since sLORETA computed the smoothest possible 3D distributed current source density solution constrained to grey matter, this approach is particularly suited for our analyses, since it does not need an *a priori* number of focal sources. In addition, sLORETA statistically locates the main generator of the maximum EEG/ERP component within a specific interval. This does not exclude the co-existence of other generators (which, in experiments like this are typically numerous), but the tool highlights always the main source among the many activated in a specific interval. Therefore, only the cortical area with greater cerebral activation was found in both groups for each condition of electrical stimulation (Under Threshold, Threshold and Over Threshold) by performing separated two-tailed *t* test between ERP responses corresponding to each intensity of electric pulse and those associated to the control condition (i.e., the only entirely somatosensory and often undetected condition) in the time intervals corresponding to P1 (40–50 ms), N1 (80–90 ms) and P2 components (190–220 ms). These within-groups analyses allowed us to locate the sources of each ERP component separately in sitting controls and BR participants: evidence of between-groups differences were found from the direct comparison of the sources underlying P1, N1 and P2 components in both groups. Instead, the between-groups source analysis usually provides the location of the maximum difference between controls and BR participants in the selected time interval, a method which does not allow to locate the effective generator of the selected ERP component within each group. The maximum difference source location is a popular and useful procedure, but it might locate a generator far from the real source of the EP component. For this reason, we preferred to carry out three within-groups analyses (Under Threshold, Threshold and Over Threshold vs. Control intensity) for P1, N1 and P2 components separately in control and BR groups, and then we discussed about the implication for differences in source locations.

## Results

### Subjective pain and anxiety reports

The between-groups *t* tests carried out on state (STAI-Y1) and trait (STAI-Y2) anxiety scores showed no significant effects (STAI-Y1 *t*(1,18) =  .38, *NS*, mean scores [± SD]: 33.60 [±8.82] vs. 32.30 [±6.18] for controls and BR participants, respectively; STAI-Y2 *t*(1,18) =  .95, *NS*, mean scores [± SD]: 37.50 [±7.68] vs. 34.80 [±4.64] for controls and BR participants, respectively). The repeated-measures ANOVA carried out on state (STAI-Y1) scores acquired before and after the whole experimental session revealed no main effects of Group and Time factors, nor their significant interaction.

Analysis of subjective pain evaluation collected during the EEG recording phase revealed different subjective judgments between groups for Threshold condition (*t*(1,18) =  1.69, *P* =  .05 one-tailed), in which reduced subjective pain perception was found in BR compared with control participants (mean pain ratings [± SD]: 2.74 [±1.11] vs. 3.57 [±1.07], respectively). A tendency to reduced subjective pain in BR compared with controls was also observed in the other pain condition (Over Threshold mean pain ratings [± SD]: 3.57 [±1.25] vs. 4.26 [±0.90] respectively, *t*(1,18) =  1.41, *P* =  .08). It could be argued that the observed differences in subjective pain evaluation reflected different basal levels of electric pain threshold between groups. However, electrical intensities corresponding to subjective pain thresholds achieved during the pain threshold assessment did not differ between groups (*t*(1,18) =  1.03, *NS*; Controls: 3.93±2.56 mA, BR participants: 2.86±2.05 mA), and subjective pain reports differed only during the next EEG recording phase.

### Electrophysiological data

#### P1 component

**Figure 3:**
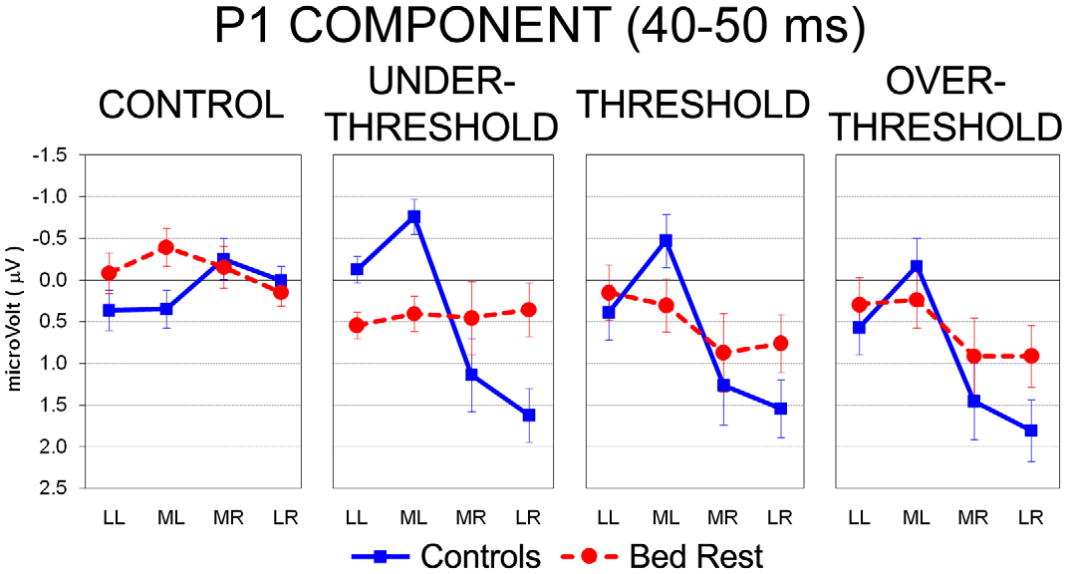
Analysis of P1 component during the 40- to 50-ms epoch after electrical stimuli: significant three-way Group by Stimulus Intensity by Laterality interaction. Mean activity and Standard Error (SE) are depicted for Control (blue bars) and Bed Rest group (red bars). Control group (blue line) showed greater positivity on right vs. left clusters of electrodes (contralateral to the side of stimulation) for Under Threshold, Threshold and Over Threshold intensities, whereas BR group (red dotted line) revealed no difference among the four stimulus conditions.

Statistics computed on the 40- to 50-ms epoch following electrical stimulation revealed a main effect of the Intensity factor (*F*(3,54) =  17.22, HF *ε* = 1.00, *P*< .001): the three pain-related conditions elicited in both groups significant greater positivity with respect to the control condition (all *P*< .001). In addition, among the three pain-related stimulations, the Over Threshold level evoked greater positivity that the Under Threshold one (*P*< .05). The Laterality main effect was also significant (*F*(3,54) = 7.08, HF *ε* =  .73, *P*< .001), showing greater positivity of both medial and lateral right locations in comparison with their respective homologues in the left hemisphere (all *P*< .05). Thus, significant greater positivity marked the cortical sites of the hemisphere contralateral to the electrically stimulated left wrist. Interestingly, the three-way Group by Intensity by Laterality interaction (*F*(9,162) = 3.02, HF *ε* =  .63, *P*< .01) revealed that only sitting controls exhibited this significant greater positivity at right locations (i.e., lateral and medial clusters) with respect to left hemisphere homologues, regardless of stimulus intensity (all *P*< .01; Fig. 3, full line). Conversely, BR subjects showed the same potentials at left and right locations, exhibiting no differences among the three pain-related levels (Fig. 3, dotted line).

#### N1 component

**Figure 4:**
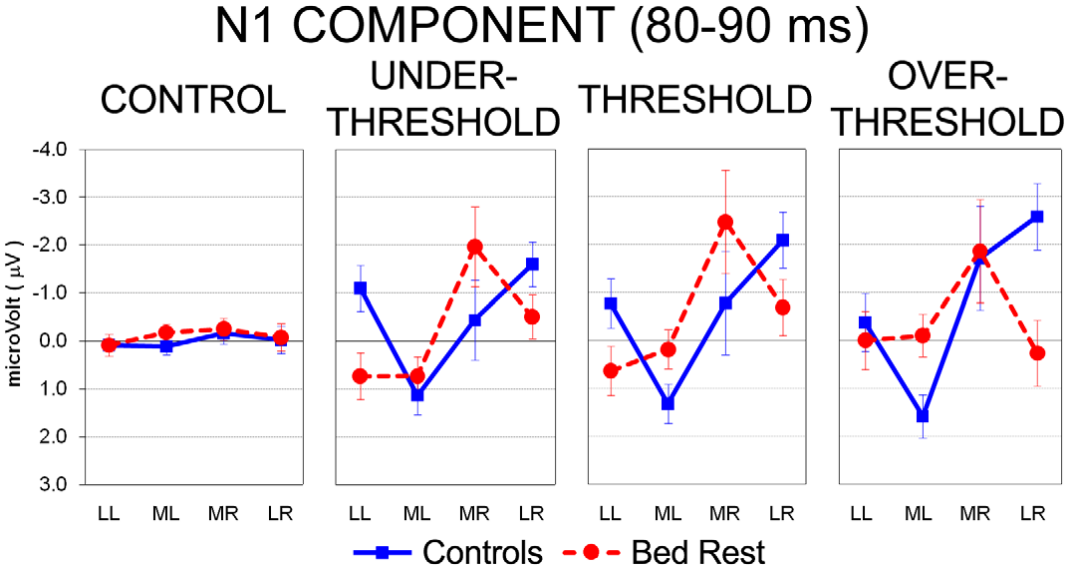
Analysis of N1 component during the 80- to 90-ms epoch after electrical stimuli: significant three-way Group by Stimulus Intensity by Laterality interaction. Mean activity and Standard Error (SE) are depicted for Control (blue bars) and Bed Rest group (red bars). During Under Threshold and Threshold intensities, control group (blue line) showed greater negativity on lateral right vs. medial left clusters of electrodes, whereas BR group (red dotted line) exhibited greater negativity on medial right vs. both left clusters. During Over Threshold condition, controls showed greater negativity in right clusters vs. medial left sites, and greater negativity than BR participants in the lateral right cluster. No between-group differences have been found in the control condition.

Similarly to the earlier time interval, ANOVA computed on the 80- to 90-ms epoch following electrical stimulation revealed a main effect of the Intensity factor (*F*(3,54) = 4.59, HF *ε* =  .62, *P*< .05), but in this window the three pain-related levels elicited greater negativity than the control one (all *P*< .01). The significant Laterality main effect (*F*(3,54)  = 5.39, HF *ε* =  .73, *P*< .01) showed greater positivity at both medial and lateral right locations in comparison with median left sites (all *P*< .01), whereas no differences were found between right and left lateral clusters. However, the three-way Group by Intensity by Laterality interaction (*F*(9,162)  = 2.79, HF *ε* =  .39, *P*< .05) showed different patterns of activation between controls and BR subjects at all pain-related levels (Fig. 4). Indeed, BR subjects showed significant greater negativity in medial right compared with the two clusters of the left hemisphere (all *P*< .01) for Under Threshold and Threshold levels, whereas controls exhibited significant greater negativity in lateral right sites compared with medial left electrodes (*P*< .01 and *P*< .001 for Under Threshold and Threshold, respectively). Instead, in correspondence of the Over Threshold level, controls showed significant greater negativity in both medial and lateral clusters of the right hemisphere compared with medial left sites (all *P*< .001; Fig. 4). In this latter intensity, groups exhibited overlapping levels of negativity at medial right locations, whereas controls had significant greater negativity than BR subjects in the lateral right cluster (*P*< .001; Fig. 4). No between group differences have been found in the control condition.

#### P2 component

**Figure 5:**
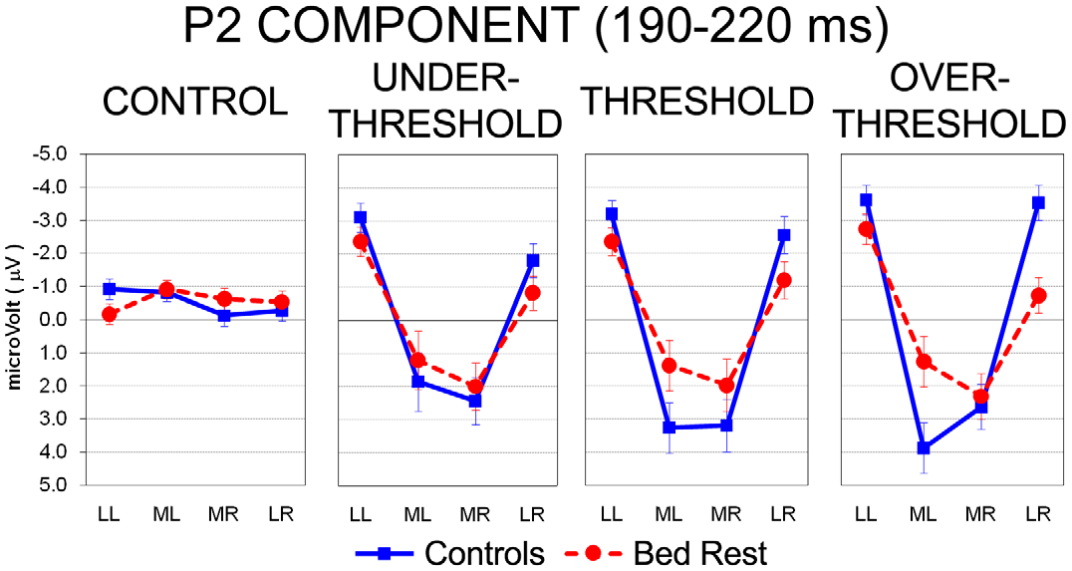
Analysis of P2 component during the 190- to 220-ms interval after electrical pulse: significant three-way Group by Stimulus Intensity by Laterality interaction. Mean activity and Standard Error (SE) are depicted for Control (blue bars) and Bed Rest group (red bars). With the exception of Control condition, both groups exhibited greater positivity in medial vs. lateral locations of both hemispheres, showing the typical, inverted U-shape pattern. Compared with BR participants (red dotted line), controls exhibited greater positivity in medial left clusters for Threshold and Over Threshold conditions (blue line).

ANOVA computed on the P2 interval (190–220 ms after electrical stimulation) revealed a main effect of the Intensity factor (*F*(3,54) = 4.05, HF *ε* =  .61, *P*< .05): compared with the painless condition, greater positivity marked all pain-related levels (all *P*< .05). The significant Laterality main effect (*F*(3,54)  = 37.80, HF *ε* =  .68, *P*< .001) showed greater positivity of medial locations (regardless of hemisphere) in comparison with the two lateral clusters (all *P*< .001). The three-way Group by Intensity by Laterality interaction (*F*(9,162) = 2.65, HF *ε* =  .39, *P*< .05) was significant and again it showed no between-group differences in the control condition (Fig. 5). Both groups exhibited significant greater positivity in medial locations of both hemispheres compared with lateral left and right sites (all *P*< .001), with a typical inverted U-shape pattern. However, compared with BR subjects, control group had significant greater positivity in the medial left cluster for Threshold and Over Threshold levels (*P*< .05 and *P*< .001, respectively), and only during the Over Threshold level, BR subjects exhibited relatively greater positivity than controls over lateral right sites (*P*< .001; Fig. 5).

### Source analyses

**Figure 6:**
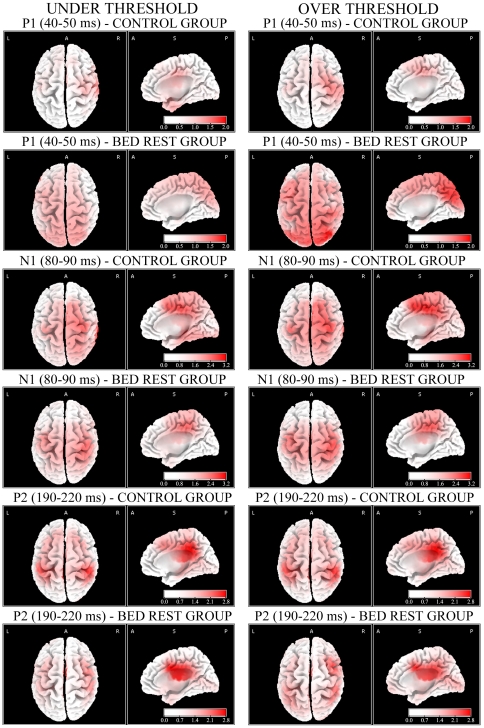
Source localization computed with sLORETA for Under and Over Threshold conditions (left and right column, respectively) for control and BR groups during P1 (first and second row, respectively), N1 (third and fourth row, respectively) and P2 components (fifth and sixth row, respectively). In the first and third columns are depicted the top views of source analyses, in the second and forth ones the midsagittal views.

Concerning the first positive component P1, corresponding to the 40–50 ms time interval, in the control group significant greater positivity was found for Under Threshold, Threshold and Over Threshold pain levels with respect to control-painless stimuli (all *P*< .05). sLORETA analyses located the source of this early positive wave, both for Under Threshold and Threshold levels, in the rostral portion of the right postcentral gyrus and, for Over Threshold level, in the caudal portion of the right postcentral gyrus (Table 1; Fig. 6, first row for Under and Over Threshold levels). Analyses carried out in BR sample revealed again significant greater positivity for Under Threshold (*P*< .05), Threshold and Over Threshold pain levels (all *P*< .01) with respect to control-painless stimulus. However, brain sources were found in the left temporopolar area/periamygdaloid cortices for both Under and Threshold levels, but within the right superior parietal lobule for Over Threshold level (Table 1; Fig. 6, second row for Under and Over Threshold levels).

**Table 1:** Source analyses of the first positive component P1 (40–50 ms) in controls and BR participants.

P1 COMPONENT (40–50ms)						
CONTROL GROUP						
Pain intensity	BA	Name	MNI coords			*t* test
			x	y	z	
Under Threshold	3	rostral postcentral gyrus	45	−25	40	*P*< .05
Threshold	3	rostral postcentral gyrus	25	−15	50	*P*< .05
Over Threshold	2	caudal postcentral gyrus	40	−30	40	*P*< .05

BR GROUP						
Pain intensity	BA	Name	MNI coords			*t* test
			x	y	z	
Under Threshold	38	temporopolar area	−40	20	−35	*P*< .05
Threshold	38	temporopolar area	−40	14	−40	*P*< .01
Over Threshold	7	superior parietal lobule	30	−65	50	*P*< .01

Each sLORETA brain source was obtained from the within-group comparison of painless, control stimuli (corresponding to a virtual zero condition of somatosensory stimulation) with Under Threshold, Threshold or Over Threshold conditions. Cortical activities elicited by each of these three latter stimuli were all significant, and their main generators were all located in the postcentral gyrus.

BA  =  Brodmann Area; MNI coords  =  Montreal Neurological Institute coordinates.

In control sitting subjects, the first negative component N1, corresponding to the 80–90 ms time interval, showed significant components at all pain-related intensities compared with painless control condition (all *P*< .01). sLORETA analysis located the source of N1 component, for the Under Threshold condition, again in the right postcentral gyrus and, for both Threshold and Over Threshold levels, in the right ventral anterior cingulate areas (Table 2; Fig. 6, third row, Under and Over Threshold levels). Analyses carried out in BR subjects revealed significant greater negativity for all pain-related levels compared with control-painless stimuli (all *P*< .01): electrical sources were found in the rostral portion of the right postcentral gyrus (Table 2), regardless of painful intensity (Fig. 6, fourth row for Under and Over Threshold levels).

**Table 2:** Source analyses of the first negative component N1 (80–90 ms) in controls and BR participants.

N1 COMPONENT (80–90 ms)						
CONTROL GROUP						
Pain intensity	BA	Name	MNI coords			*t* test
			x	y	z	
Under Threshold	2	caudal postcentral gyrus	55	−35	30	*P*< .01
Threshold	24	ventral anterior cingulate	15	−15	50	*P*< .01
Over Threshold	24	ventral anterior cingulate	15	−15	50	*P*< .01

BR GROUP						
Pain intensity	BA	Name	MNI coords			*t* test
			x	y	z	
Under Threshold	3	rostral postcentral gyrus	30	−35	50	*P*< .01
Threshold	3	rostral postcentral gyrus	30	−35	50	*P*< .01
Over Threshold	3	rostral postcentral gyrus	30	−30	50	*P*< .01

Each sLORETA brain source was obtained from the within-group comparison of painless control condition (corresponding to a virtual zero condition of somatosensory stimulation) with Under Threshold, Threshold or Over Threshold conditions. Cortical activities in the above-mentioned contrasts were all significant, and their main generators were located in the postcentral gyrus (control group – Under Threshold condition, and BR group – all intensities) and in the ventral portion of anterior cingulate cortex (control group – Threshold and Over Threshold conditions).

BA  =  Brodmann Area; MNI coords  =  Montreal Neurological Institute coordinates.

The second positive component P2, corresponding to the 190–220 ms time interval, showed in both groups significant greater positivity between painful and painless control conditions (Table 3). sLORETA analysis located the source of P2 component, elicited by all pain-related conditions, over the left dorsal posterior cingulate areas in controls (Table 3), and over right ventral anterior cingulate in BR subjects (Table 3; Fig. 6, last two rows for control and BR groups, respectively).

**Table 3:** Source analyses of the second positive component P2 (190–220 ms) in controls and BR participants.

P2 COMPONENT (190–220 ms)						
CONTROL GROUP						
Pain intensity	BA	Name	MNI coords			*t* test
			x	y	z	
Under Threshold	31	dorsal posterior cingulate	−15	−35	40	*P*< .01
Threshold	31	dorsal posterior cingulate	−15	−35	40	*P*< .01
Over Threshold	31	dorsal posterior cingulate	−15	−35	40	*P*< .01

BR GROUP						
Pain intensity	BA	Name	MNI coords			*t* test
			x	y	z	
Under Threshold	24	ventral anterior cingulate	10	5	40	*P*< .01
Threshold	24	ventral anterior cingulate	10	5	40	*P*< .05
Over Threshold	24	ventral anterior cingulate	15	10	40	*P*< .05

Each sLORETA brain source was obtained from the within-group comparison of painless control condition (corresponding to a virtual zero condition of nocicettive/somatosensory stimulation) with Under Threshold, Threshold or Over Threshold conditions. Cortical activities elicited by each of these three latter stimuli were all significant, and their main generators were located in the dorsal portion of the posterior cingulate cortex (control group) or in the ventral portion of the anterior cingulate cortex (BR group).

BA  =  Brodmann Area; MNI coords  =  Montreal Neurological Institute coordinates.

## Discussion

The present study aimed to investigate the effects of the microgravity – simulated with the Head-Down Bed Rest (HDBR) position – on pain-related somatosensory processing in a group of healthy adults matching characteristics of astronauts. During the EEG experimental session, in which participants had to estimate different levels of electrical painless/painful stimuli, BR subjects underestimated pain intensities in comparison to sitting controls in the pain Threshold condition, revealing a reduced subjective sensitivity to pain as a consequence of the bed rest position. Interestingly, considering early electrophysiological components (P1) peaking at about 45 ms, sitting controls showed greater activation in both medial and lateral right sites compared to the left ones, i.e., contralaterally to the side of stimulation, regardless of stimulus intensity (Fig. 3). Conversely, BR subjects exhibited reduced cortical modulations, which did not differentiate activity among the four locations. Past studies on somatosensory evoked potentials showed that the P45 component typically represents the neural activity in primary somatosensory (SI) cortex contralateral to stimulation side [42]–[46], therefore the lack of significant P45 component contralaterally to the stimulus side in BR subjects may be interpreted as an inhibited cortical somatosensory processing induced by bed rest condition. Source analysis made with sLORETA helps to clarify statistical results achieved from electrode clustering, nevertheless it is important to be cautious in its interpretation as this program provides only one main electrical generator for each analysis. This does not exclude the parallel contribution of other sources (which are typically involved in an extended neural network on pain processing) not marked by the program, but that secondarily contribute to the overall scalp activity and to results obtained from electrode clusters. Analyses carried out with sLORETA located the source of P45 component in controls' right postcentral gyri (BAs 3-2; Fig. 6, first row), in agreement with past literature on early ERP components which suggested that waves peaking before 80 ms reflect the earliest brain responses to incoming somatosensory information [1]). In BR subjects, the source of P45 elicited by Under Threshold and Threshold levels was located in left temporopolar cortex, whereas in Over Threshold condition P45 source was located in right superior parietal lobule (Fig. 6, second row). These results suggest that, compared with controls, bedridden participants had dampened response of the main source in the somatosensory cortex which probably unmasked other sources related to the interaction of the electrical stimulus with the unpleasant head-down condition [19]. Indeed, the activation of temporopolar region (which is very close and connected with the amygdaloid complex) for painless or pain threshold intensities suggests that these stimuli are not completely processed at central level, at least in this early interval. This could depend on a fast subcortical pathway connecting sensory thalamus to the amygdala [47], [48]. It is currently accepted that the amygdala, together with the hippocampus and surrounding cortices (e.g., entorhinal cortex), is part of an extended pain network and contributes to the affective-aversive components of pain [see 5,49 for reviews, but also 50–54]. Concerning the activation of the left temporopolar cortex in the BR group rather than in the Control group, one plausible explanation is that HDBR is a moderately unpleasant position [19], characterized by perceived face swelling, and this might have triggered the temporal-emotional component of the pain network. This small source should be activated also in the later components of the BR group, but since LORETA provides only the first main generator, for the N1 and P2 the strongest dominant activity is located more centrally, closer to the main sources found also in controls. Differently from the two smaller electrical stimulations, the Over Threshold stimulation in BR group activated the right superior parietal lobule, an associative region close to the stimulated somatosensory cortex, and typically involved in spatial attention orienting [e.g. 55], [56] – a result which suggests the capability of higher electrical intensities to induce a significant activation of the attention orienting system, possibly aimed at automatic locating the stimulated skin area.

The analysis of late cortical potentials, i.e., N1 and P2, supposed to reflect all phases of painful/painless stimulus processing, from the detection of its basic characteristics in somatosensory cortices (SI-SII) to top-down cognitive aspects of pain detection [1], [3], showed altered patterns of cortical activation in BR subjects compared with controls. Indeed, concerning the first negative component (i.e., N1) peaking about 85 ms after electrical stimuli, BR subjects exhibited significant greater activation in right medial locations, contralateral to the side of stimulation, whereas controls showed significant greater activation of the right lateral sites and reached the maximum during the Over Threshold level (Fig. 4). Analyses computed with sLORETA in this time interval located controls' source of N1 in the postcentral gyrus, when stimulation intensity was low and painless, but in the ventral portion of the anterior cingulate cortex (corresponding to the BA 24) when pain intensities reached and exceeded individual threshold levels (Fig. 6, third row). This finding is in agreement with past neuroimaging studies which related different subregions within the ACC (BA 24) to subjective pain sensations and affective component of pain [e.g. 5], [57], [58], to the shift of attention to painful stimulus [e.g. 59]–[61] and to the integration of all affective and cognitive aspects of pain anticipation, learning and empathy [e.g. 10], [62]–[64]. In line with this interpretation, only the highest, most aversive, electrical stimulation was able to activate anterior cingulate and affective dimension of pain [5]. Instead, BR participants exhibited a pattern of cortical processing in the primary somatosensory area: similarly to Controls' P1 component, in N1 interval significant greater activation was found in the postcentral gyrus, regardless of stimulus intensity (Fig. 6, fourth row). Therefore, somatosensory tactile-related processing mediated by Aβ fibers [1], [3] occurred in primary somatosensory area of BR participants 80–90 ms after stimulus onset, while controls showed, in correspondence of the strongest stimuli (Threshold and Over Threshold conditions), the shift of activity to the anterior cingulate possibly related to an increased orienting of attention to painful stimuli and the processing of their affective aspects.

Concerning the second positive component (i.e., P2) peaking at 190–220 ms, both groups exhibited similar pattern with greater medial cluster activation (Fig. 5). In this time window, however, controls exhibited greater positivity than BR subjects in left medial sites for both Threshold and Over Threshold conditions (Fig. 5). In addition, increased intensity of electrical stimulation evoked greater central positivity in controls but not in BR subjects, who showed reduced undifferentiated levels of activation regardless of electrical intensity. Analyses computed with sLORETA located the source of P2 in controls' posterior cingulate cortex, mainly in the dorsal portion corresponding to the BA 31, whereas in BR subjects P2 source was found in the anterior cingulate cortex, mainly in the ventral portion corresponding to the BA 24 (Fig. 6, last two rows). Also for the P2 component, BR participants showed a different pattern of cortical pain-related somatosensory processing with respect to sitting controls: the main source of this component (in the Threshold and Over Threshold pain intensities) was, in BR subjects, in the ventral anterior cingulate, the same area which in Controls was activated in correspondence of the N1 component.

These results are very important particularly considering that they have been found in a sample of young healthy adults after a relative short-term period of 2 hours in the HDBR position. Given the technical limitation of medical interventions in space environment, the consequences of such impaired pain-related somatosensory perception in astronauts could delay the detection of severe illnesses and interfere with ambitious long-term space missions. However, from a very different point of view, the present electrophysiological data raise an issue on possible implications for bedridden patients. Indeed, a long-term hospitalization, in which patients are confined to bed, could significantly alter the cognitive and perceptual functioning, and particularly pain-related somatosensory processing. Accordingly, an undetected pain signalling, e.g., a cardiac stroke or an internal haemorrhage, may induce wrong diagnosis of life-threat diseases, and could be fatal to the untreated patient. Bedridden patients usually lie for long time on the bed and they are often elderly patients with age-related cognitive decay: thus, future investigations aimed at clarifying bedridden patients' pain processing could help to improve their medical treatment and to prevent dangerous mental and physical degradation. Concerning the variables which could link the head-down body position with cortical pain-related somatosensory inhibition, among the many possible candidates (as written in the introduction almost all physiological indexes, including cardiovascular ones, are affected by microgravity) arterial baroreceptors and their bottom-up cortical neural projections are the most probable [65], [66]. Past studies have shown how also limited stimulation of baroreceptors is able to dampen pain cortical responses [67], [68]. However, the causal or intermediate variables which could play a role in the observed pain dampening is a matter of future studies.

In conclusion, the present study provided evidence of two important issues related to effects of simulated microgravity on pain-related somatosensory processing. First, the component with a latency of 45 ms, representing the earliest brain response to incoming strong electrical stimulation and located in contralateral somatosensory cortices was substantially altered in BR participants, revealing reduced subjective and cortical somatosensory processing. Second, late components with latencies of 85 and 200 ms, which account for all phases of painful/painless stimulus processing, from the detection of its basic characteristics in somatosensory cortices (SI-SII) to top-down cognitive aspects of pain perception, showed in BR subjects no pain modulations across three electrical levels. Furthermore, compared with Controls, BR group showed a significant delay in the activation of pain-specific areas. This study highlighted, for the first time, the possible implications of altered pain perception and cortical pain-related somatosensory processing induced by short-term HDBR position for astronauts and, more generally, for long-term bedridden patients.
